# Pathology of the Uterus and Its Appendages

**Published:** 1835-04-01

**Authors:** 


### Pathology of the Uterus and its Appendages. By *Robert Lee*, M.D. F.R.S.

Among the clever articles in the Cyclopædia of Practical Medicine, Dr. Lee's paper, under the above title, holds a distinguished station. The pathological researches of this talented and indefatigable physician are beyond all praise, and must, ere long, place him high on the list of benefactors to the human race, and cultivators of the healing art. Of this article, which, of course, is elementary, we can only give a general outline—and that of only certain prominent portions.

Dr. Lee divides the diseases of the uterus into three kinds—1, those produced by inflammation--2, by the formation of tumors not malignant—and 3, by malignant action, disorganizing its different textures.

Inflammation of the lining membrane of the uterus sometimes produces merely an increase of the natural secretion—sometimes a puriform discharge. In painful menstruation, there is occasionally thrown off, as is now well known, a kind of membrane, something like the decidua. Such females usually suffer uneasiness in the region of the uterus, even in the intervals of menstruation. "The false membrane is probably formed between the monthly periods, by a peculiar and specific inflammation of the mucous coat of the uterus. The symptoms would lead to the inference that the substance of the uterus is affected." Since the time of Morgagni, (who first described this disease,) most authors have acknowledged the obstinacy of the complaint. Denman recommended mercury to ptyalism, with ammoniated tincture of iron, various tonics, and Tunbridge waters, &c. with indifferent success. Burns said that time was more frequently efficacious than physic. Dewees exhibited camphor and gum arabic to relieve the pain; and the volatile tincture of guaiacum as a more permanent remedy. The pathology and treatment, however, of this affection are far from being settled. The occasional detraction of blood from the pelvic region during the intervals of menstruation, is useful; while the exhibition of calomel, Dover's powder, and camphor—tepid injections—and the horizontal position, will be necessary also. The complaint is nearly allied to what Dr. Gooch has denominated "irritable uterus." In this doctrine Dr. Dewees does not agree.

"Chronic congestion and inflammation of the uterus appear to arise most frequently from exposure to cold and fatigue during menstruation, and subsequent to abortion or parturition. It is an obstinate disease, and often resists the effects of all remedies for many months or even years. The patient should remain in the horizontal position if the pain is constant and severe. Blood must be drawn from the arm or from the uterine region by leeches, or by cupping-glasses. When the circulation is undisturbed, as is most frequently the case, local is to be preferred to general bloodletting. Some think that cupping affords decidedly more relief than leeches, and that the glasses should be applied over the sacrum, or to the part to which the pain is referred. Dr. Dewees applies them to the inside of the thighs. The bowels should be regulated by castor-oil, infusion and electuary of senna, super-tartrate of potash, and Epsom salts. To subdue the pain the tepid hip-bath, warm fomentations, and narcotics must be had recourse to, and camphor combined with extract of hyoscyamus, henbane, or poppy, should be administered twice or thrice a day. A belladonna plaster should be applied over the sacrum. Warm decoction of poppy, or lukewarm linseed tea, or eight or ten grains of opium dissolved in a pint of hot water, or solution of starch, may be thrown up the vagina, and an ounce of warm milk with a drachm of laudanum may be injected into the rectum, after the bowels have been evacuated. An alterative course of mercury has afforded decided relief in some cases. Like all the other chronic phlegmasiæ, when the disease has lasted long, relief sometimes follows a different plan of treatment, viz. the employment of exercise, bitters, tonics, sulphurous and chalybeate waters. Where the stomach has suffered much, the phosphate of iron may be given with advantage.

Chronic inflammation of the uterus does not degenerate into cancer, as many suppose, and it rarely terminates in suppuration of the muscular tissue of the uterus. Cases of abscess of the walls of the unimpregnated uterus have been described by writers, but they are very seldom met with. Mr. Howship has a uterus in his possession, in the muscular coat of which, or in the cellular membrane between its layers, was an abscess which contained about an ounce of pus. The symptoms were not ascertained before death. This is the only example of abscess of the walls of the uterus from simple inflammation that we have seen; those abscesses in the uterus described by Dr. Hooper were connected with malignant disease of the organ. Where a collection of pus has taken place within the cavity of the uterus, there has also in most cases been present a malignant organic affection of the os and cervix uteri." 9.

Dr. Lee makes some observations on inflammation of the follicles of the os uteri—or, as Mad. Boivin calls it—granular inflammation of the os uteri—a disease little understood, and only to be detected by the speculum.

"The os uteri is swollen, red, ecchymosed, morbidly sensible when touched, and disposed to bleed. There is often present a leucorrhœal discharge from the vagina, and a state of excitement bordering upon nymphomania. In some cases the affection has been misunderstood, from the absence of local symptoms, or because it has been accompanied with more severe lesions. The granulations, when hard, are usually very small, like grains of sand or the seeds of the poppy; if they are larger, their softness prevents them from being discovered, except by a very experienced practitioner.

These granulations are found in a subacute or chronic state. In the former they are seen on the lips of the os uteri, sometimes in small numbers like peas, firm and white; more frequently in great numbers, like grains of millet-seed, also white and soft, and vesicular, without roots. It is from their interstices that the blood flows which escapes into the vagina when they are touched, or when the bowels are evacuated.

In the chronic state the enlarged follicles or granulations are hard, small, and white, and rest on soft, red, miliary elevations, in one case like varicose veins. The causes of this affection are not the same in all cases; they are often obscure, like the causes of all uterine diseases. In some cases, the affection seems to have been produced by syphilis or some cutaneous disease, or by the presence of a fibrous tumour in the uterus. In the examination of dead bodies we have repeatedly seen the appearances described by Boivin, and we agree with her in thinking that they depend on an enlargement of the mucous follicles of the os uteri. We have seen numbers of these bodies much enlarged, both in the vagina and os uteri, when individuals had died from chronic disease unconnected with any morbid state of the uterus.

Emollients and local bloodletting are the remedies recommended by Boivin in the subacute stage of the disease. The treatment must be stimulating in the chronic stage, and afterwards, in the greater number of cases, derivatives must be had recourse to. The greatest advantages have resulted from their use in many cases. Where the disease is syphilitic, mercury must be employed." 10.

Tympanitis of the uterus is next treated of, but Dr. Lee acknowledges that no instance of this or of dropsy of the unimpregnated uterus has ever come under his observation. They are rare diseases.

Our author proceeds to the consideration of tumors and enlargements situated in the orifice of the uterus, not of a malignant character. These he divides into the fibrous, the follicular or granular, the cystic or vesicular, and the mucous tumors. He has published an important paper on this subject in the second part of the 18th volume of the Medico-Chirurgical Transactions ; but as we devoted much space to this subject in our last number, we must be brief in the present instance.

" The fibrous tumour is usually of a globular form, and varies greatly in size. It has generally a cartilaginous and fibrous structure, and the fibres are often disposed in a concentric or converging manner. This tumour has sometimes a granular appearance, or seems to consist of a congeries of smaller tumours, of different densities, each having a thin capsule of cellular membrane. When large, the tumour is often unequal, lobulated, or divided by deep fissures, and arteries and veins of considerable magnitude can be traced into its substance. Cavities containing a bloody or dark-coloured gelatinous fluid are sometimes formed in the central parts of the tumour, by a process of softening which its substance undergoes. In other cases the tumour does not manifest a disposition to become softer as it enlarges, but its density gradually increases until the whole mass has become cartilaginous, without arteries or veins containing red blood ; or calcareous depositions are gradually formed in the substance of the tumour, until it is partially or completely converted into a concretion of phosphate or carbonate of lime. This is generally of a light yellow colour or nearly white, soft and porous, like pumice-stone ; but instances have occurred where it has become so hard as to admit of being polished like marble or ivory. These deposits usually first take place in the most dense points of the tumour. In a few rare cases, they have been formed on the surface of the tumour, and have inclosed it like the shell of an egg. Gardien states that the smallest tumours most frequently undergo this transformation. Andral, on the authority of Brugnatelli, states that carbonate and phosphate of lime, with an animal or gelatinous matter, enter into the composition of these bodies. Dr. Turner, professor of chemistry in the London University, had the kindness to analyse, at our request, two years ago, a small concretion, which was passed during life from the uterus of a female above sixty years of age. This was found to consist entirely of carbonate of lime and animal matter. Dr. Bostock has more recently analysed several specimens of uterine concretions, and he has found them principally to consist of phosphate and carbonate of lime with animal matter.*

In several cases of fibro-calcareous tumour of the uterus which have come under our observation, and of which we have related the histories in the paper referred to, little uneasiness was experienced during life; but in another case there was also malignant ulceration of the uterus, and portions of the calcareous tumour were discharged from the vagina long before the disease proved fatal. Many months previous to her death this patient had attacks of hæmorrhage and excruciating pains in the uterus before the concretions were passed. There were also sallowness of the complexion, and great irritability of stomach, as in cases of malignant disease." 13.

---

* Med Chirurg. Transact. vol. xviii. part ii. p. 313.

Where these tumors are detected by examination, and where they hang loose, they may be removed by a pair of forceps. When inflammation of the uterus results from the presence of these bodies, leeches and other appropriate means should be employed.

"The history of fibrous tumors and polypi of the uterus" occupies several columns of the Cyclopædia, and is very ably drawn up by Dr. Lee. It is incapable of abridgement; but it will be read with interest and profit by the numerous subscribers to that meritorious work.

### Treatment.

"When formed under the peritoneum and between the muscular fibres of the uterus, fibrous tumours are but little under the influence either of external or internal remedies. Iodine and mercury have little effect either in arresting their growth or promoting their absorption. The increased determination of blood which often takes place to the uterus when these bodies are formed in its walls, should be relieved by local bloodletting, anodynes, and rest in the recumbent position; and when profuse hemorrhage occurs, it should be controlled by rest in the recumbent position, cold applications to the hypogastrium, the tampon, and the superacetate of lead. The uneasy sensations from pressure on the bloodvessels and nerves of the lower extremities may sometimes be slightly relieved by certain changes of posture; and if the tumour be moveable and occupies the hollow of the sacrum, and compresses the bladder and rectum, it may be removed from this situation by pressing it above the brim of the pelvis. In most cases fibrous tumours cannot be removed by art while they remain within the cavity of the uterus. When the hemorrhage endangers life, some authors recommend us to dilate the os uteri artificially, and to remove the tumour. Lisfranc has recorded a case in which incisions were made through the os uteri and the tumour removed.

When fibrous tumours are formed under the lining membrane of the uterus, and have passed through its orifice into the vagina, constituting polypi, they may be removed by a ligature, or their root may be divided with a knife, or they may be twisted off. Since the invention of the double canula by Levret, various instruments have been employed for passing ligatures around the stems of uterine polypi. For polypi of ordinary dimensions the instrument of Gœrz, improved by Niessen and Dr. Gooch, is the best that can be employed. When the tumour is of large dimensions, a curved rod or tube is preferable. When the two silver canulæ are made use of, a strong ligature must be introduced through both tubes, so that its two ends may hang out of their lower apertures, while the middle portion forms a noose between the two upper apertures. Thus armed, the canula must be passed over the globular part of the tumour, the fore-finger of the left hand having previously been introduced as a guide to the instrument. One of the tubes is then to be kept fixed, while the other tube is to be carried slowly round the circumference of the root of the tumour until it reaches the opposite side of the tube, which has been kept in the same place. The ligature must be tightened until the neck of the tumour is completely cut across. When the tumour becomes putrid, and many days elapse before its root is divided by the ligature, the tumour should be drawn down, and the peduncle should be divided with the knife or scissors. The greatest attention should be paid to cleanliness, and the offensive discharge should be washed away by injection of tepid water and solutions of the chlorurets. This operation is not without danger. In a case which occurred in St. George's Hospital, under the care of Mr. Babington, the patient died of uterine phlebitis. M. Blandin saw a case terminate fatally from the same disease. Cases have repeatedly terminated unfavourably from ulceration being excited in that part of the uterus to which the tumour had adhered. Dupuytren states that he has met with eight or ten cases where patients were destroyed after the application of a ligature around the root of a polypus of the uterus, and where the symptoms were those produced by the absorption of pus into the system. M. Dupuytren has removed two hundred uterine polypi by excision in the course of the last twenty years. In this large number hemorrhage has only taken place twice, and in both these instances it was permanently arrested by plugging. In eight cases M. Velpeau has never met with hemorrhage. Many other distinguished continental surgeons prefer the excision of uterine polypi to their removal by the ligature, and our experience inclines us to prefer the former method. Where the root of the tumour is largely supplied with bloodvessels, as in a recent case which came under our observation, to obviate the danger of hemorrhage after its division, a ligature should previously be firmly applied around it, at a short distance from the uterus. Dubois affirms that even this does not secure the patient from hemorrhage. Dupuytren seizes the tumour with the forceps of Museux, and draws it down till the os uteri can be seen at the entrance of the vagina; a pair of curved scissors is then conducted along the finger to the root of the tumour and it is divided. It is only in cases where the neck of the polypus is slender and of soft consistence that it can be safely twisted off." 21.

We pass over the malignant or cancerous diseases of the uterus—for, unfortunately, "there are no means by which we can prevent or remove them." They do not depend on a common inflammation, but a specific action of the parts, which proceeds invariably, sooner or later, to destruction of the patient. Still the sufferings of the individual may often be mitigated by guarding against those attacks of plethora and inflammation which frequently occur in the course of the malady, and aggravate the symptoms. Leeches to the labia pudendi are the best means on such occasions. Battley's liq. opii sed. introduced into the rectum, with a small quantity of thin starch, will often relieve pain when opium given by the mouth fails.

An instructive chapter is occupied with diseases of the vagina and vulva, followed by a section on those of the urethra, forming a good epitome of our knowledge on these topics. It is incapable of analysis. We return Dr. Lee our best thanks for the information contained in this part of the Cyclopædia.

